# First record of the adult of *Cymbalcloeon* confirming its position in Baetidae (Insecta, Ephemeroptera)

**DOI:** 10.3897/BDJ.13.e178464

**Published:** 2025-12-22

**Authors:** Ningning Wang, Di Wang, Jean-Luc Gattolliat, Chanaporn Suttinun, Changfa Zhou

**Affiliations:** 1 Nanjing Normal University, Nanjing, China Nanjing Normal University Nanjing China; 2 Naturéum, Lausanne, Switzerland Naturéum Lausanne Switzerland; 3 Lausanne University, Lausanne, Switzerland Lausanne University Lausanne Switzerland; 4 Chiang Mai University, Chiang Mai, Thailand Chiang Mai University Chiang Mai Thailand

**Keywords:** China, morphology, origin, phylogeny, systematics

## Abstract

**Background:**

The nymphs of *Cymbalcloeon sartorii* Suttinun et al., 2020 present several unusual characters such as having only three pairs of ventral gills. The imaginal stage of this recently described genus remains unknown.

**New information:**

In Spring 2025, tens of nymphs as well as morphologically and molecularly associated adults of this species were collected in southwestern China. This represents the first recorded occurrence of the genus in China, and indeed, the first report outside Thailand. The male imagos have tiny bodies, clear median longitudinal ridge on mesonotum, one pair of wings without any marginal intercalary veins, relatively long apical segment of styliger, and distinct penis cover. The morphology of this species shows it is close to *Baetopus* Keffermüller, 1960 in the subfamily Cloeoninae. However, it lost some key structures, like marginal intercalaries, hindwings and most gills.

## Introduction

The genus *Cymbalcloeon* and the type-species *Cymbalcloeon sartorii* Suttinun et al., 2020 were described by Suttinun et al. (2020) based on nymphal specimens collected in Thailand. The nymphs are characterized by three pairs of ventral gills on abdominal segments V–VII, suggesting that *Cymbalcloeon* represents a highly derived clade within the family Baetidae. In addition to the unique gill structure, the nymphs of *C. sartorii* exhibit distinctive apomorphies. All three pairs of legs with tibio-patellar sutures and the mandibles lacking setae between the molar and incisor regions are key characters that define distinct lineages within Baetidae, according to Kluge (1997). Other notable features include two-segmented maxillary and labial palpi, the absence of spines along the lateral margins of the terga, the presence of two rows of teeth on the claws, and clearly divided compound eyes. In this context, the discovery and description of the adult stage of the genus are of great significance for understanding its phylogenetic placement.

Various attempts were proposed to classify the Baetidae; the family was divided into two (Kazlauskas 1972), subsequently in three (Gillies 1991) and finally in five subfamilies (Kluge 1997) (including a fossil subfamily). However, this kind of classification was questioned (Gattolliat et al. 2008, Nieto 2010, Cruz et al. 2020), and the key character of their divisions (single or double marginal intercalary veins) was already doubted by Riek (1973) and Morihara and Edmunds (1980) as well as most of the subsequent authors. Recent discoveries of additional genera provided valuable insights into the origin and evolution of the Baetidae (Gillies 1990, Kluge and Suttinun 2020); however, genetic analyses and character-based studies are essential to fully resolve the global phylogeny of this highly diverse family.

Riek (1973), Landa and Soldán (1985) and Kluge (1997), Kluge (2022), placed the family Siphlaenigmatidae or subfamily Siphlaenigmatinae next to true Baetidae. However, the family Siphlaenigmatidae does not have marginal intercalaries or turban eyes, but it has distinct penes and its forceps have two apical segments instead of one as in Baetidae. Molecular phylogeny confirms the basal position of Baetidae within the order Ephemeroptera but challenges its previously proposed sister relationship with Siphlaenigmatidae (Ogden et al. 2009, Ogden et al. 2019). Identifying the most basal genera within Baetidae is crucial for understanding its evolutionary relationships with other families and for bridging existing phylogenetic gaps.

In March 2025, we conducted a mayfly collection in Yunnan Province, southwestern China. During this survey, we collected two *Cymbalcloeon* nymphs along with several imagos that were tentatively associated with them. To obtain additional material for molecular confirmation of the association between stages, we returned to the site in May 2025. The molecular analysis confirms the conspecificity of the nymphs and adults. This allows us to report, for the first time, the adult stage of *Cymbalcloeon*, providing new insights into the origin and evolutionary history of the family Baetidae. This also represents the first occurrence of the genus in China, and the first report outside of Thailand.

## Materials and methods

### Specimen collection and image processing

The nymphs were collected by hand net in a small stream, the imagoes and subimagoes were attracted by light-traps. All materials were stored in ethanol (more than 80%). All specimens used in this study are deposited in the Mayfly Collection, College of Life Sciences, Nanjing Normal University (NNU).

Most structures are observed directly in ethanol (about 80%). Two genitalia are dissected from male and dissolved in NaOH (10%) for 10 minutes at 40°C.

Digital photos were taken by Sony a7R (Interchangeable Lens Digital Camera), and some of them were taken under Nikon Eclipse 50i (Microscope) and Mshot MZ81 (Stereomicroscope). Final plates were prepared with Adobe Photoshop 2022.

Eggs were dissected from the female subimago. All SEM samples were dehydrated in graded acetone solutions (70%, 80%, 90%, 2×98% and 2x100%) for 10 min each. Subsequently, the specimens were immersed in 1-1.5 mL HMDS (1,1,1,3,3,3 hexamethyldisilazane; Merck-Suchardt, Darmstadt) in 20 mL glass vials. After a soak of 30 minutes, approximately 90% of the HMDS was removed and the vials were immediately transferred to a desiccator. The bottom of the desiccator was covered by silica gel beads (Merck-Suchardt, Darmstadt）and the desiccator itself was vacuumized. The remaining HMDS was allowed to evaporate overnight under anhydrous conditions. Afterwards, the specimens were sputter coated with gold (BIO-RAD SC510, München) for 80 seconds. The samples were photographed by a Scanning Electron Microscope (Apreo 2S, Thermo Fisher Scientific Company, Waltham, MA, USA).

### COI sequence

Total DNA was extracted from one nymph and two male subimagos of *C. sartorii* using Animal Genomic DNA Kit (TsingKe Biotech Co., Beijing, China). The mitochondrial gene cytochrome c oxidase subunit I fragment was PCR-amplified using the Premix Taq (Takara Bio Inc., Beijing, China), PCR conditions included initial denaturation at 94 °C for 5 min, 40 cycles of denaturation at 94 °C for 30 s, annealing at 50 °C for 30 s, and extension at 72 °C for 40 s, with a final extension at 72 °C for 10 min. Universal COI primers were referenced from Zheng and Zhou (2021). Sequences were aligned using Muscle, and the K2P genetic distances within genus were calculated in MEGA11. The two obtained sequences have been uploaded to GenBank (Table 1). CO1 sequences were compared against reference sequences in the NCBI GenBank and BOLD (Barcode of Life Data System) databases to assess similarity and potential species-level matches.

## Taxon treatments

### Cymbalcloeon (Sartorii) sartorii

Suttinun et al. 2020

B4FF006F-40FC-5040-A653-42720EF67CC2

#### Materials

- **Type status:** Other material. **Occurrence:** recordedBy: De-Wen Gong, Xu-Hong-Yi Zheng, Ning-Ning Wang, Yu-Xian Sun; individualCount: 2; lifeStage: nymph; occurrenceID: E6970792-99D4-5D80-9234-FAF9B25FC7CD; **Taxon:** scientificName: *C. sartorii*; **Location:** country: China; stateProvince: Yunnan; county: Mengla; locality: Man-Nan-Xing, Mengla County, Xishuangbanna Dai Autonomous Prefecture, Yunnan Province, China; verbatimElevation: 530m; verbatimCoordinates: 21°53.00′N 101°17.00′E; decimalLatitude: 21.8921; decimalLongitude: 101.288; **Event:** eventDate: 2025-03-18/20
- **Type status:** Other material. **Occurrence:** recordedBy: De-Wen Gong, Xu-Hong-Yi Zheng, Ning-Ning Wang, Yu-Xian Sun; individualCount: 15; sex: male; lifeStage: imago; occurrenceID: 86F631A9-6C54-5B4E-A9E5-416B5740C879; **Taxon:** scientificName: *C. sartorii*; **Location:** country: China; stateProvince: Yunnan; county: Mengla; locality: Man-Nan-Xing, Mengla County, Xishuangbanna Dai Autonomous Prefecture, Yunnan Province, China; verbatimElevation: 530m; verbatimCoordinates: 21°53.00′N 101°17.00′E; decimalLatitude: 21.8921; decimalLongitude: 101.288; **Event:** eventDate: 2025-03-18/20
- **Type status:** Other material. **Occurrence:** recordedBy: De-Wen Gong, Xu-Hong-Yi Zheng, Ning-Ning Wang, Yu-Xian Sun; individualCount: 2; sex: female; lifeStage: imago; occurrenceID: 0092A0E4-8941-5670-A3A5-453F160CED6B; **Taxon:** scientificName: *C. sartorii*; **Location:** country: China; stateProvince: Yunnan; county: Mengla; locality: Man-Nan-Xing, Mengla County, Xishuangbanna Dai Autonomous Prefecture, Yunnan Province, China; verbatimElevation: 530m; verbatimCoordinates: 21°53.00′N 101°17.00′E; decimalLatitude: 21.8921; decimalLongitude: 101.288; **Event:** eventDate: 2025-03-18/20
- **Type status:** Other material. **Occurrence:** recordedBy: De-Wen Gong, Xu-Hong-Yi Zheng, Ning-Ning Wang, Yu-Xian Sun; individualCount: 20; sex: male; lifeStage: subimago; occurrenceID: A60DB51B-8EE6-52E1-B8EA-48183513F517; **Taxon:** scientificName: *C. sartorii*; **Location:** country: China; stateProvince: Yunnan; county: Mengla; locality: Man-Nan-Xing, Mengla County, Xishuangbanna Dai Autonomous Prefecture, Yunnan Province, China; verbatimElevation: 530m; verbatimCoordinates: 21°53.00′N 101°17.00′E; decimalLatitude: 21.8921; decimalLongitude: 101.288; **Event:** eventDate: 2025-03-18/20
- **Type status:** Other material. **Occurrence:** recordedBy: De-Wen Gong, Xu-Hong-Yi Zheng, Ning-Ning Wang, Yu-Xian Sun; individualCount: 3; sex: female; lifeStage: subimago; occurrenceID: 12437BA3-835A-5BA1-AD82-486B5E8225B4; **Taxon:** scientificName: *C. sartorii*; **Location:** country: China; stateProvince: Yunnan; county: Mengla; locality: Man-Nan-Xing, Mengla County, Xishuangbanna Dai Autonomous Prefecture, Yunnan Province, China; verbatimElevation: 530m; verbatimCoordinates: 21°53.00′N 101°17.00′E; decimalLatitude: 21.8921; decimalLongitude: 101.288; **Event:** eventDate: 2025-03-18/20
- **Type status:** Other material. **Occurrence:** recordedBy: De-Wen Gong and Di Wang; individualCount: 10; lifeStage: nymph; occurrenceID: 18207030-8E64-52DD-806C-3C35976AA8B3; **Taxon:** scientificName: *C. sartorii*; **Location:** country: China; stateProvince: Yunnan; county: Mengla; locality: Man-Nan-Xing, Mengla County, Xishuangbanna Dai Autonomous Prefecture, Yunnan Province, China; verbatimElevation: 530m; verbatimCoordinates: 21°53.00′N 101°17.00′E; decimalLatitude: 21.8921; decimalLongitude: 101.288; **Event:** eventDate: 5/10/2025

#### Description

**Figure 1.**
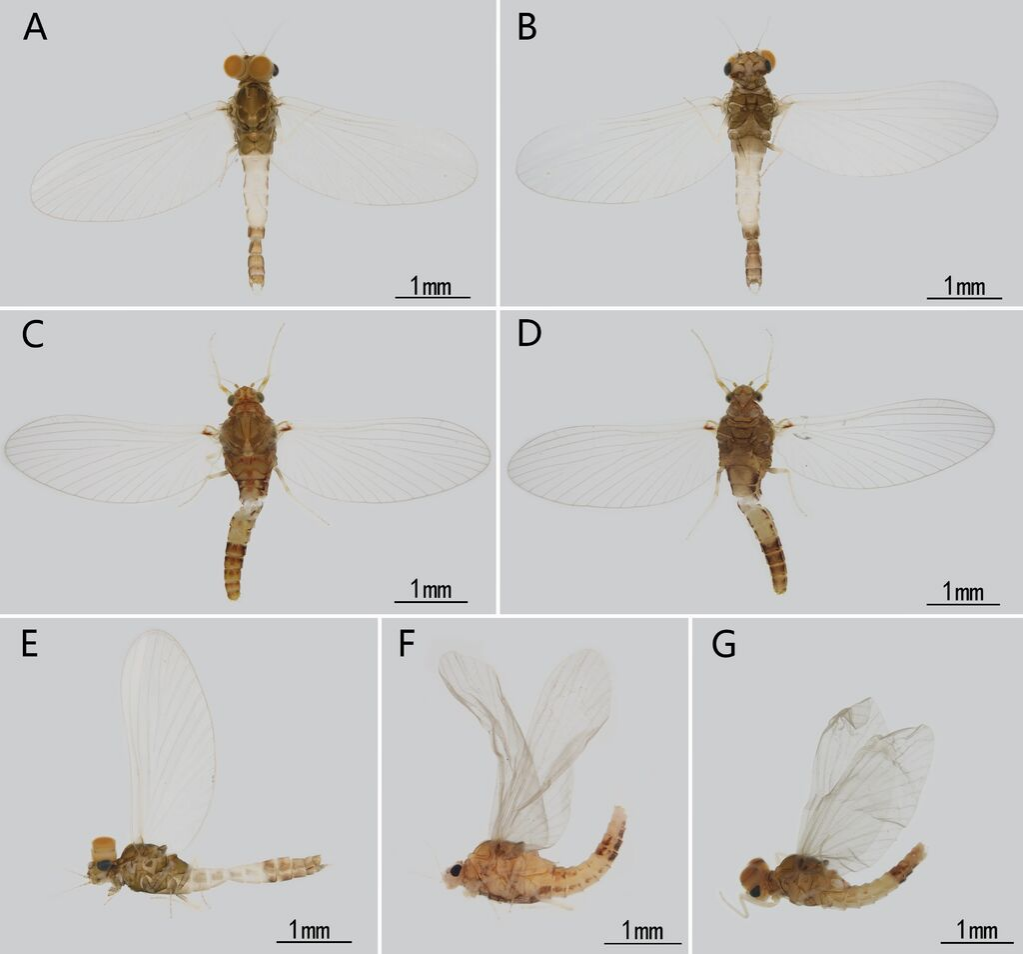
Imaginal habitus of *C. sartorii*. **A** male (dorsal view); **B** male (ventral view); **C** female (dorsal view); **D** female (ventral view); **E** male (lateral view); Subimaginal habitus of *C. sartorii*: **F** female subimago (lateral view); **G** male subimago (lateral view).

Male imago (in alcohol). Body length 3.4 mm, forewing 3.0 mm, cerci 3.6 mm (Fig. 1A, B, E).

**Figure 2.**
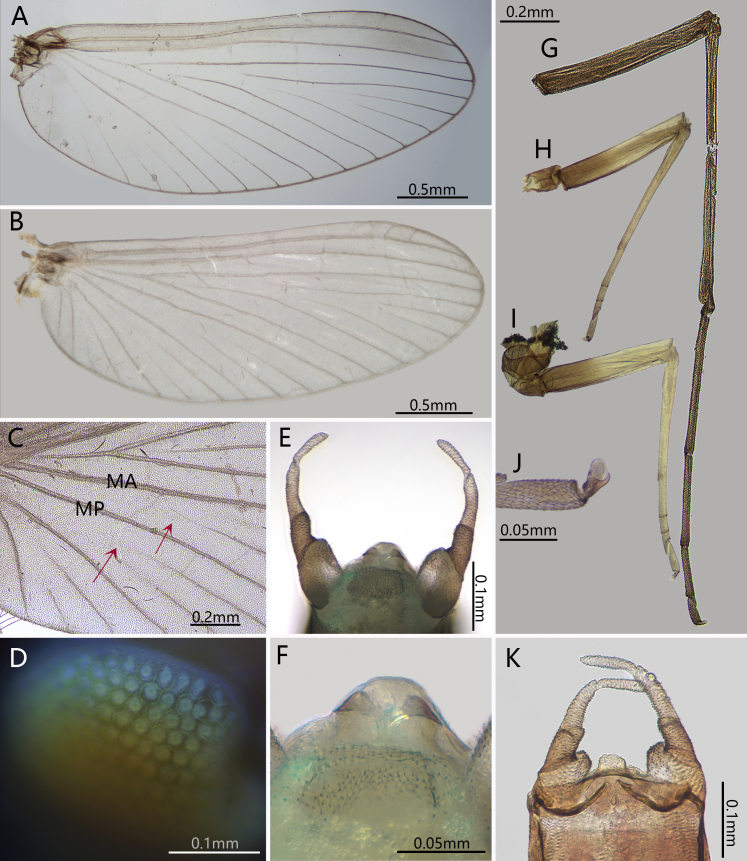
Imaginal characters of *C. sartorii*. **A** forewing of male; **B** forewing of male subimago; **C** forewing of male subimago enlarged (arrows indicating the forking points of MA₁ & MA₂ and MP₁ & MP₂); **D** compound eyes; **E** genitalia (ventral view); **F** subgenital plate; **G** foreleg of male; **H** midleg of male; **I** hindleg of male; **J** claw of hindleg; **K** genitalia (dorsal view, showing the penial bridge).

Head. Light reddish brown (Fig. 1A, B). Scape and pedicel cylindrical, light brown, flagella light brown to whitish, ca. 6.5x pedicel in length, antennal length equivalent to head width (Fig. 1A, B). Dorsal surface of upper portion of compound eyes orange, lateral surface pale grey (Fig. 1E); upper portion of eyes cylindrical (Fig. 1E); lower portion black, semi-spherical (Fig. 1E); facets of compound eyes hexagonal (Fig. 2D). Ocelli with brown base but pale apex (Fig. 1A).

**Figure 3.**
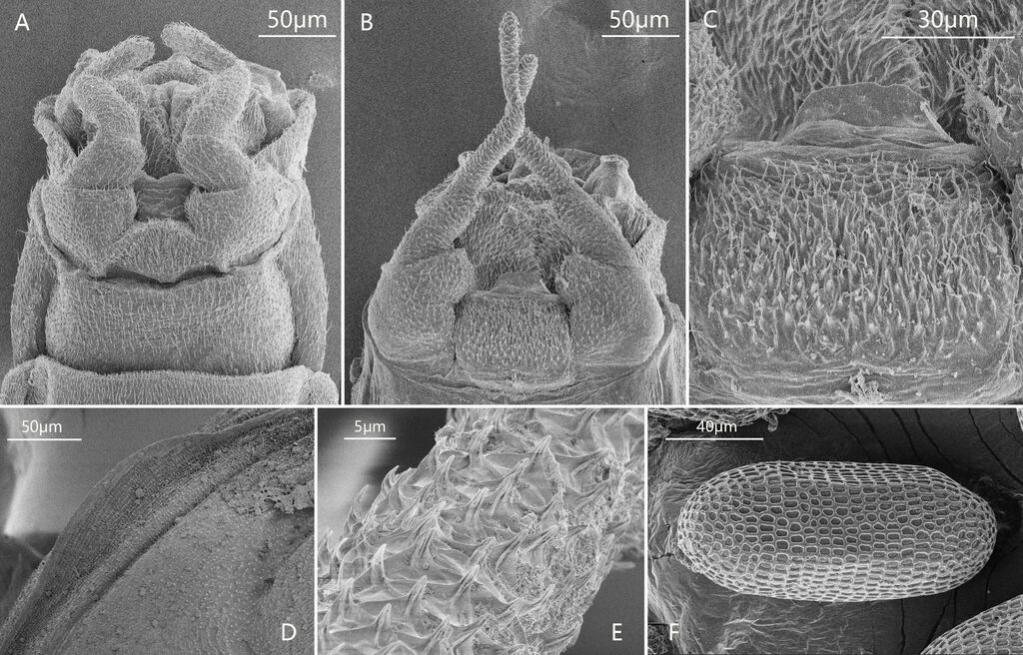
SEM photos. **A** genitalia of male subimago; **B** genitalia of male imago; **C** subgenital plate of male imago; **D** mesonotum of male imago; **E** tarsus of hindleg; **F** egg from female subimago.

Thorax. Nota of thorax brown to deep brown but with pale sutures (Fig. 1A) with distinct longitudinal median ridge on mesonotum (Fig. 1E, Fig. 3D). Scutella of meso- and metanota expanded into projections (Fig. 1E).

Foreleg pale brown, length ratio of forefemur: tibia: tarsus=1.0: 1.5: 1.7; length ratio of segments of foretarsus=1.0: 7.5: 4.9: 3.0: 1.4; foreleg with two claws, one acute, one blunt (Fig. 2G). Basal half of midfemur brown, other part light brown, tibia and tarsus light brown (Fig. 2H). Length ratio of midfemur: tibia: tarsus=2.5: 3.3: 1.0; length ratio of three free segments of midtarsus =1.3: 1.0: 2.3; one claw acute, one claw blunt (Fig. 2H). Basal half of hindfemur brown, other part light brown (Fig. 2I). Length ratio of hindfemur: tibia: tarsus=2.5: 3.2: 1.0; length ratio of three segments of hindtarsus=1.1: 1.0: 2.0 (Fig. 2I). Claws similar to fore- or midlegs (Fig. 2J).

Costal and subcostal veins of forewings light brown, other longitudinal veins whitish to light brown (Fig. 2A); crossvein greatly reduced, only 4-5 crossveins between C and Sc, 3-4 between Sc and R_1_, two between other neighbored longitudinal veins (Fig. 2A). MA_2_ and MP_2_ detached from MA_1_ and MP_1_ respectively (Fig. 2A–C). Membrane of forewing transparent (Fig. 2A). Sc, Rs_1_ and Rs_5_ with bullae; CuP with a back-curved base but most part of it and A_1_ straight (Fig. 2A).

Abdomen. Segment I, VI-X reddish brown, other segment pale with dark brown stripes on lateral margins of terga; terga I-VI with a pair of submedian reddish dots respectively, but in most cases, these dots indistinct to invisible; terga VII-X with a pair of additional submedian stripes respectively (Fig. 1A, B, E). Cerci pale.

Genitalia: Pedestal of gonostylus distinctly wider than gonostylus (forceps) (Fig. 2E, K, Fig. 3B); gonostylus with two segments, basal half of segment I slightly widened; segment II with slightly broadened apex, its length subequal to half segment I (Fig. 2E, K, Fig. 3B). Subgenital plate with tiny hair-like setae on surface (Fig. 3B, C). Penis cover distinct, nearly quadrate in shape (Fig. 2K). Gonovectes or penial arms smoothly curved (Fig. 2K).

Female imago (in alcohol). Body length 2.9 mm, forewing 3.0 mm, cerci broken (Fig. 1C, D). Similar to male imago but with more reddish pigments, especially thorax and abdomen (Fig. 1C, D). Length ratio of forefemur: tibia: tarsus=1.2: 1.1: 1.0; length ratio of segments of foretarsus=1.0: 2.8: 1.7: 1.7: 2.1 (Fig. 1C, D). Midlegs broken. Hindlegs similar to forelegs in color (Fig. 1C, D). Length ratio of hindfemur: tibia: tarsus=2.0: 3.2: 1.0; length ratio of segments of hindtarsus=1.1: 1.5: 1.0 (Fig. 1C, D).

Male subimago: (in alcohol). Body length 3.1 mm, forewing 2.4 mm, cerci 4.4 mm (Fig. 1G). Length ratio of forefemur: tibia: tarsus=1.5: 1.0: 1.7; length ratio of midfemur: tibia: tarsus=1.8: 1.6: 1.0; length ratio of segments of hindtarsus=3.4: 4.1: 1.0 (Fig. 1G). Veins of forewing slightly clearer than male, MA_2_ and MP_2_ connected to MA_1_ and MP_1_ with a crossvein respectively (Fig. 2B). Genitalia distinct, gonostyliger and segment II of styliger oriented inwards but segment I of styliger oriented outwards; length of them: segment I > segment II > gonostyliger (Fig. 3A).

Female subimago: (in alcohol). Body length 2.7 mm, forewing 2.9 mm, cerci 4.0 mm (Fig. 1F). Length ratio of forefemur: tibia: tarsus=1.5: 1.4: 1.0; Length ratio of midfemur: tibia: tarsus=2.0: 2.0: 1.0; Length ratio of hindfemur: tibia: tarsus=3.5: 3.9: 1.0 (Fig. 1F).

**Figure 4.**
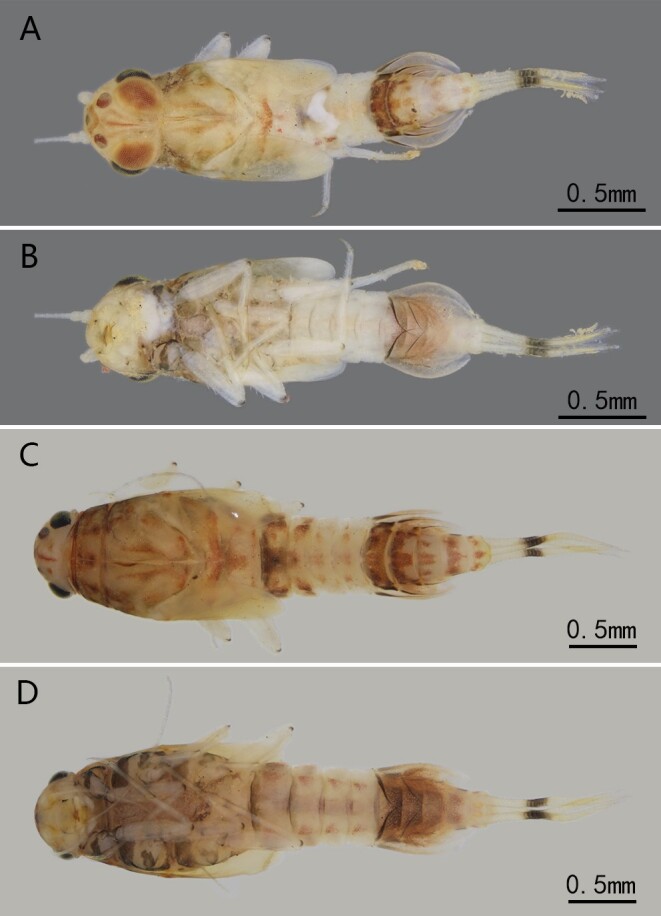
Nymphal habitus of *C. sartorii*. **A** male nymph (dorsal view); **B** male nymph (ventral view); **C** female nymph (dorsal view); **D** female nymph (ventral view).

**Figure 5.**
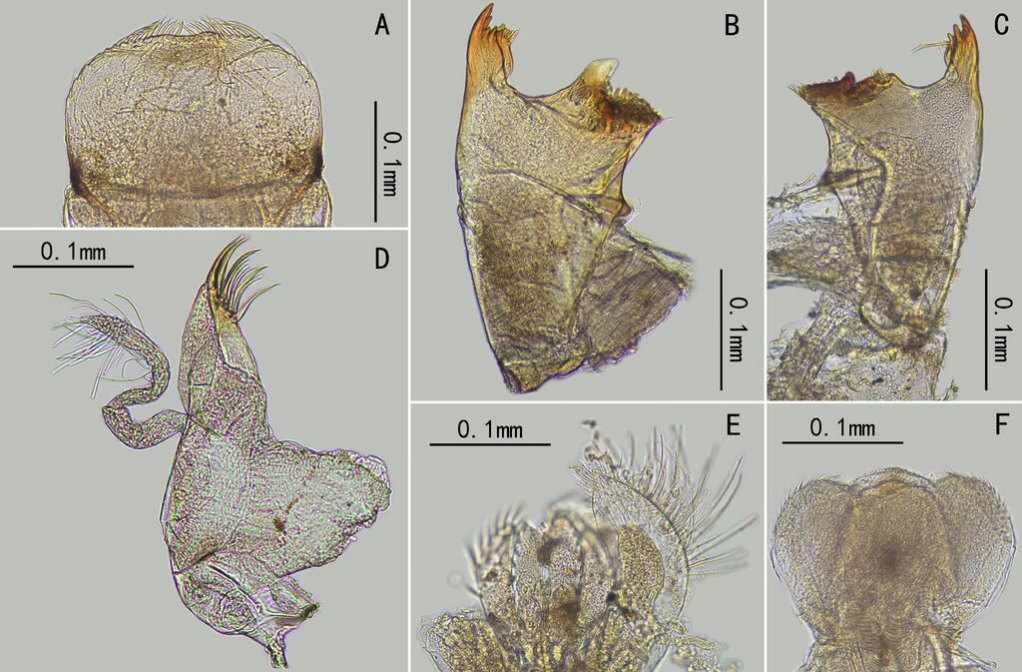
Mouthpart of *C. sartorii*. **A** labrum; **B** left mandible; **C** right mandible; **D** maxilla; **E** labium; **F** hypopharynx.

**Figure 6.**
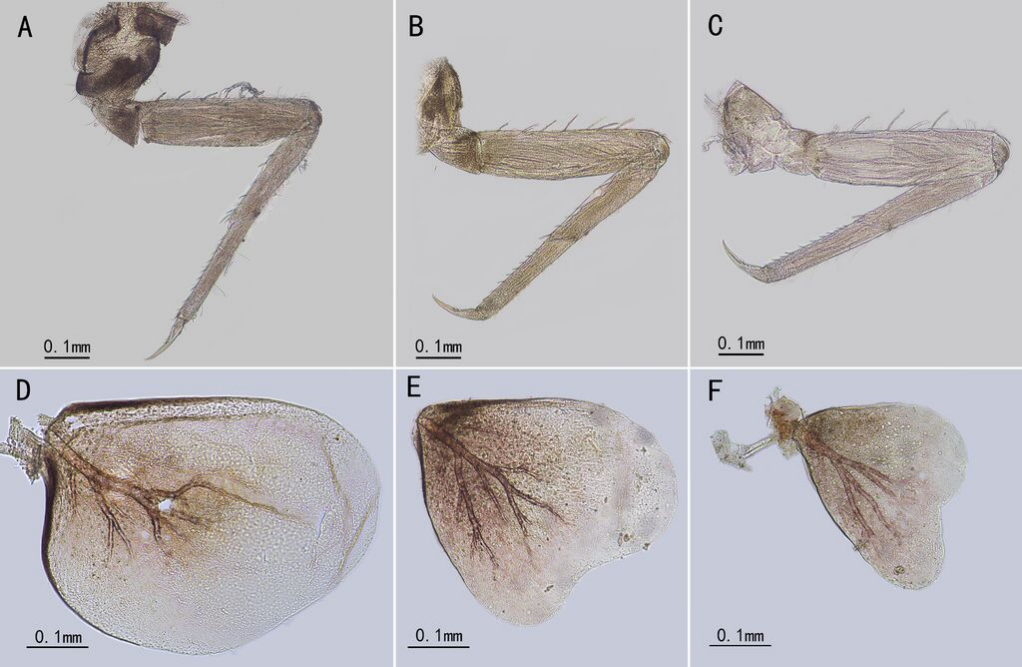
Nymphal characters of *C. sartorii*. **A** foreleg; **B** midleg; **C** hindleg; **D** Gill V; **E** Gill VI; **F** Gill VII.

Nymph (in alcohol) (see Suttinun et al. 2020 and Figs 4, 5, 6).

The main characteristics including: Body length 2.5 mm, cerci 1.3 mm (Fig. 4A–D). Body color pattern similar to adults, pale body with reddish dots and streaks; thorax distinctly broader and more robust than abdomen (Fig. 4A–D). Mouthparts shown in figure 5, labrum with a very shallowly concave anterior margin (Fig. 5A); mandibles without any setae between mola and incisor, outer margin without setae, prostheca with spiny apex (Fig. 5B, C); maxillae with spines on apex of galealacinia, maxillary palpi two segmented, apical segments with hair-like setae (Fig. 5D); labium with long crescent glossae and paraglossae (Fig. 5E); labial palpi two-segmented, apex of segment II expanded into a small knob-like structure (Fig. 5E). Hypopharynx with very short hair-like setae on dorsal surfaces (Fig. 5F). Tibiae and tarsi of all legs subequal in length, each of them ca. 0.8x femora (Fig. 6A–C); outer margin of femora and inner margins of tibiae and tarsi with spine-like setae (Fig. 3E). Gills on abdominal segment V-VII, gills I nearly oval, with deeply pigmented margins; gills II and III with concaved posterior margin (Fig. 6D–F). Caudal filaments with a distinct dark band (Fig. 4).

Egg: length 126.4-131.6 µm, width 54.4-58.3 µm, long oval, whole chorion decorated with irregularly-shaped ridges (from quadrate to oval) (Fig. 3F). The area surrounded by ridges concave, forming tiny pits (Fig. 3F).

#### Nymph and adult association

Nymphs and adults in the present study were associated by two methods. Morphologically, we use color pattern and body size, particularly those reddish patches on abdomen (both terga and sterna of segment VI-X are pigmented), to identify and pair them. Molecularly, we sequenced COI gene of one nymph and two male subimagos. The data shows the K2P genetic distance is lower than 0.3%. Sequence comparison did not identify any close relatives in public genetic repositories. The closest one is a baetid sequence in GenBank, the genetic distance between them is 15.1%.

## Discussion

Although the adults of *C. sartorii* do not have marginal intercalary veins, a key diagnostic character of the family Baetidae, we still place this species in the Baetidae. The reasons are: (1) it has turban upper portion of compound eyes (Fig. 1E); (2) the longitudinal venation of the forewings typical of Baetidae (Fig. 2A, B); (3) its genitalia is similar to some *Afroptilum* Gillies, 1990 and *Indocloeon* Müller-Liebenau, 1982 (see Kluge and Suttinun 2020), without visible penes (Fig. 2E, F, Fig. 3B, C); (4) mid- and hindtarsi have three movable segments (Fig. 2H, I); (5) the very base of CuP bent backward, which is regarded as an apomorphy of the Turbanoculata sensu Kluge (1997)(=Baetidae) (Fig. 2A, B); (6) its nymphal mouthparts are similar to common baetids, like mandibles without lateral setae and fused incisors, and the shape of maxillae and labium (Fig. 5A–F); (7) Y-shaped epicranial suture reaching ventrally of lateral ocelli.

Based on known nymphs at that time, Suttinun et al. (2020) thought the monospecific genus *Cymbalcloeon* is possible close to *Baetopus* Keffermüller 1960 or *Raptobaetopus* Müller-Liebenau 1978 (see *Baetopus* in Bauernfeind and Soldán 2012). The first imaginal morphology described here shows this relation is correct. First, *Cymbalcloeon* and *Baetopus* share similar forceps or gonostyli (two segmented, segment II subequal to half segment I) (Fig. 2E, K). Second, they have similar penis cover (nearly quadrate) (Fig. 2K). Third, body size is tiny, just 3–5 mm in length (Figs 1, 4). Although *Cymbalcloeon* has neither marginal intercalaries in forewings nor hindwings (Fig. 2A, B), it is believed here to be a close clade to the genus *Baetopus* in the subfamily Cloeoninae. In nymphal stage, the mouthparts, legs, claws and body shape of *Cymbalcloeon* and *Baetopus* are similar too (Figs 4, 5, 6). Whole egg surface of them is covered with ridges (Fig. 3F).

Three characters of *Cymbalcloeon* are remarkable: nymphs with three pairs of gills on abdominal segments V-VII (Fig. 6D–F), imagos without hindwings and marginal intercalaries in forewings (Fig. 2A, B). However, in the family Baetidae, the loss of the hindwing happened in several lineages, and the loss of marginal intercalaries was also found in several taxa, like unknown baetids reported by Gillies 1991. So those two characters (without hindwing or margical intercalaries), we think, are collateral results of its body reduction. The nymphal morphology, in particular their peculiar gills, shows that *Cymbalcloeon* occupies a unique niche in the aquatic ecosystem. During the collecting, the nymphs of this species were observed clinging to and moving on the pebble surface, their abdomens swinging swiftly; these characteristics are not found in other baetids.

## Supplementary Material

XML Treatment for Cymbalcloeon (Sartorii) sartorii

## Figures and Tables

**Table 1.** The species and their COI sequences used in this research.

Species	GenBank accession number	Remarks
*C. sartorii*	PV800332	China (Yunnan), Nymph
*C. sartorii*	PV800333	China (Yunnan), Male subimago
